# Molecular Reports of Ruminant *Babesia* in Southeast Asia

**DOI:** 10.3390/pathogens11080915

**Published:** 2022-08-14

**Authors:** Eloiza May Galon, Iqra Zafar, Shengwei Ji, Hang Li, Zhuowei Ma, Xuenan Xuan

**Affiliations:** National Research Center for Protozoan Diseases, Obihiro University of Agriculture and Veterinary Medicine, Obihiro 080-8555, Hokkaido, Japan

**Keywords:** *Babesia*, molecular epidemiology, PCR, cattle, water buffalo, goat, sheep, Southeast Asia, tick-borne

## Abstract

The protozoon *Babesia* is a blood parasite transmitted by hard ticks and commonly parasitizes ruminants such as cattle, buffaloes, goats, and sheep. Babesiosis, the disease caused by *Babesia* infection, has been considered a potential threat to ruminant production due to the grave and enormous impact it brings. About 125 million ruminants are at risk of babesiosis in Southeast Asia (SEA), a region composed of 11 countries. In recent decades, molecular-based diagnostic platforms, such as polymerase chain reaction (PCR) assays, have been a reliable and broadly employed tool in *Babesia* detection. In this article, the authors compiled and summarized the molecular studies conducted on ruminant babesiosis and mapped the species, including *B. bovis*, *B. bigemina*, *B. ovata*, *Babesia* sp. Mymensingh, *Babesia* sp. Hue, and *B. ovis*, and determined the host diversity of ruminant *Babesia* in SEA.

## 1. Introduction

*Babesia* is a genus of apicomplexan parasites which parasitizes various hosts ranging from avian to domesticated and wild mammals and humans [1]. Since its first discovery over 130 years ago, greater than 100 species of *Babesia* have been reported and described, with several novel species documented only in recent years [2]. As *Babesia* is transmitted by ixodid hard ticks, its geographical distribution closely resembles that of its tick vectors, which more frequently exist in the tropical and subtropical regions of the world. Babesiosis refers to the disease caused by infection with *Babesia* parasites. Babesiosis causes hemolytic anemia, fever, inappetence, jaundice, and hemoglobinuria in animals with acute clinical disease, which could be fatal in severe cases. As one of the major tick-borne diseases (TBDs) in animals, babesiosis is a major concern due to its sizeable impact on farmers from the millions worth of direct and indirect losses to livestock production [3]. 

Breakthroughs in molecular biology have permitted the development of molecular tools which enable rapid and precise diagnosis of economically important diseases such as babesiosis. However, the lack of epidemiological data on babesiosis hinders the accurate evaluation of damages brought by this disease to ruminant farming, especially in countries where veterinary services and resources are either inaccessible or unavailable. More importantly, the scant information impedes the establishment of appropriate and adequate treatment and prevention measures against babesiosis. To this end, this mini-review aims to collate the molecular reports and map the *Babesia* species infecting cattle, water buffaloes, goats, and sheep in Southeast Asia, particularly those that utilized molecular detection tools (PCR-based assays), and to uncover insights into the available molecular information that may be useful in formulating disease control programs against babesiosis.

## 2. Livestock in Southeast Asia and Relevance of Babesiosis

**Figure 1:**
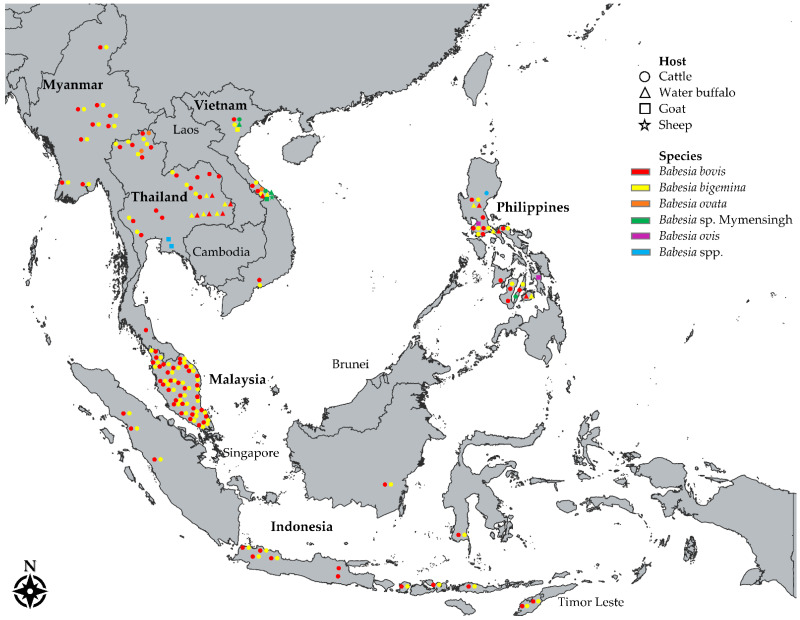
A map showing the distribution of molecularly confirmed *Babesia* parasites in ruminants across Southeast Asia. Names of countries with documented *Babesia* molecular reports in cattle (○), water buffaloes (△), goats (□), and sheep (☆) are in bold font. Each color corresponds to a species —red: *Babesia bovis*; yellow: *B. bigemina*; orange: *B. ovata*; green: *Babesia* sp. Mymensingh; purple: *B. ovis*; blue: *Babesia* spp. Each shape indicates detection of a species in a particular province or area.

Southeast Asia (SEA), a geographical region in Asia, is composed of two subregions: (a) the mainland or continental subregion, composed of Cambodia, Laos, Myanmar, Thailand, Vietnam, and Singapore, and (b) the maritime or insular subregion, composed of the archipelagos, Philippines and Indonesia, the Malaysian peninsula, Singapore, Brunei Darussalam, and Timor-Leste (Figure 1) [4]. SEA’s climate is monsoonal, characterized by wet and dry seasons, which brings plenty of rainfall to support the growing of crops and the raising of animals for food production. Agriculture accounts for a huge portion of the economy of the majority of countries in SEA, and the region is a key player in the world agro-food trade, shown by its continuously increasing agricultural exports [5].

The traditional livestock raising in SEA is based on the mixed crop-livestock systems, where the majority of ruminant farms are owned by smallholder farmers who implement either extensive or semi-intensive management practices [6]. In 2019, there were a total of 125,358,751 ruminants in SEA [7]; 43.66%, 10.89%, 30.19%, and 15.43% of the total ruminant population were cattle, water buffaloes, goats, and sheep, respectively (Table 1). More than 70% of the ruminant population is raised in Indonesia and Myanmar, while 21% can be found in Vietnam, the Philippines, and Thailand (Table 1). 

Driven by the increasing population, expanding urbanization, and higher incomes in Southeast Asia, the demand for livestock produce will continue to rise in the coming decades [8]. People in SEA derive a huge portion of their protein intake from pork and poultry meat. However, with infectious diseases such as African swine fever [9] and avian influenza [10] drastically reducing the production of the more preferred pork and poultry meat, respectively, increased consumption of alternatives such as meat from cattle (beef), water buffalo (red beef and carabeef), goat (chevon), and sheep (mutton and lamb) is highly expected. For instance, the global demand for beef is expected to increase by 25 million tons in the year 2030, with half of the projected increased annual consumption coming from Asia [11]. Consequently, this amplified demand necessitates more efficient production practices, such as minimizing potential losses through the establishment of control of economically devastating infectious diseases such as TBDs.

Several TBDs have been confirmed to be present in SEA. The warm and humid climate of SEA supports the proliferation and dissemination of tick vectors and the tick-borne pathogens they carry [12]. Regardless of the threat to the livestock industry through losses and damages, TBDs continue to be neglected, especially in resource-constrained areas. The burden of livestock babesiosis is among the gravest of the TBDs, particularly in highly susceptible herds, i.e., exotic breeds and naïve older animals, in locations where the disease is not endemically stable [13]. Alongside the efforts of some Southeast Asian countries to boost production through the introduction of improved stocks [14,15,16], either by importing purebred exotic animals or crossbreeding native species with the imported breeds, is the risk of increased susceptibility to *Babesia* infection. Thus, surveillance and monitoring are crucial in ensuring healthy livestock herds and shielding them against the adverse impacts of babesiosis.

## 3. Applicability of PCR Assays for the Detection of *Babesia* in Ruminants

The past half-century has witnessed the influx of the development of novel molecular tools which revolutionized the diagnosis of parasitic diseases. As such, molecular diagnostics has become instrumental in uncovering the epidemiology of diseases that are important in the medical, veterinary, and economic sense [17]. Likewise, a multitude of previously unknown pathogens have been discovered through the application of molecular techniques.

Unlike tools that directly detect the presence of parasites (blood smears) or determine the exposure of an animal to the parasite (enzyme-linked immunosorbent assay, indirect fluorescent antibody test, and immunochromatographic test), nucleic-acid-based diagnostic assays provide highly accurate detection of the parasite DNA in samples collected from the field, addressing the various sensitivity and specificity issues of the formerly mentioned tools [18]. Among the nucleic-acid-detecting platforms, the polymerase chain reaction (PCR) assays, including variants such as conventional PCR (cPCR), nested PCR (nPCR), and multiplex PCR assays (mPCR), have been excellent in qualitatively confirming the presence of *Babesia* through the amplification of a DNA fragment in blood samples [19].

The earliest molecular survey of *Babesia* in SEA was performed in Vietnam two decades ago [20]. Since then, several PCR-based assays have been used in subsequent studies to confirm the presence of ruminant *Babesia* in SEA, the most common of which are listed in Table 2. All documented studies used cPCR, nPCR, or mPCR assays to confirm the presence of *Babesia* DNA in blood samples of cattle (Table 3), water buffaloes (Table 4), and small ruminants (Table 5). In addition, the molecular markers targeted to detect the *Babesia* parasites have been consistent across different SEA countries, attesting to the applicability of these assays in the field. The 18S rRNA gene, along with various genes of protein families of spherical body protein, apical membrane antigen, and rhoptry-associated protein, were the most frequently targeted markers for the PCR detection of ruminant *Babesia* in SEA (Table 2).

**Table 2:** Commonly used PCR assays in detecting *Babesia* in ruminants in Southeast Asia.

Organism	Target Gene	PCR Assay Type	Target Size (bp)	Primers (5′→> 3′)	References
*Babesia bigemina*	Apical membrane antigen-1 (*ama-1*)	Nested PCR	738	GTATCAGCCGCCGACCTCCGTAAGT	[31]
				GGCGTCAGACTCCAACGGGGAACCG	
			211	TACTGTGACGAGGACGGATC	
				CCTCAAAAGCAGATTCGAGT	
	Rhoptry-associated protein-1a (*rap-1a*)	Nested PCR	879	GAGTCTGCCAAATCCTTAC	[34]
				TCCTCTACAGCTGCTTCG	
			412	AGCTTGCTTTCACAACTCGCC	[35]
				TTGGTGCTTTGACCGACGACAT	
	18S rRNA	Conventional PCR	689	TAGTTGTATTTCAGCCTCGCG	[36]
				AACATCCAAGCAGCTAHTTAG	
*Babesia bovis*	Rhoptry-associated protein-1 (*rap-1*)	Conventional PCR	356	CACGAGCAAGGAACTACCGATGTTGA	[27]
				CCAAGGACCTTCAACGTACGAGGTCA	
	Spherical body protein-2 (*sbp-2*)	Nested PCR	1236	CCGAATTCCTGGAAGTGGATCTCATGCAACC	[32]
				ATCTCGAGTCACGAGCACTCTACGGCTTTGCAG	
			580	CGAATCTAGGCATATAAGGCAT	
				ATCCCCTCCTAAGGTTGGCTAC	
	Spherical body protein-4 (*sbp-4*)	Nested PCR	907	AGTTGTTGGAGGAGGCTAAT	[33]
				TCCTTCTCGGCGTCCTTTTC	
			503	GAAATCCCTGTTCCAGAG	
				TCGTTGATAACACTGCAA	
	Variant erythrocyte surface antigen-1α (*vesa-1α*)	Conventional PCR	166	CAAGCATACAACCAGGTGG	[37]
				ACCCCAGGCACATCCAGCTA	
*Babesia ovata*	Apical membrane antigen-1 (*ama-1*)	Conventional PCR	504	GATACGAGGCTGTCGGTAGC	[38]
				AGTATAGGTGAGCATCAGTG	
*Babesia* sp. Mymensingh	Apical membrane antigen-1 (*ama-1*)	Conventional PCR	371	TGGCGCCGACTTCCTGGAGCCCATCTCCAA	[39]
				AGCTGGGGCCCTCCTTCGATGAACCGTCGG	
*Babesia ovis*	18S rRNA	Conventional PCR	549	TGGGCAGGACCTTGGTTCTTCT	[40]
				CCGCGTAGCGCCGGCTAAATA	

The 18S rRNA is an evolutionarily conserved gene and is the usual target gene for molecular detection due to its structural and functional stability, low substitution rates, and lack of horizontal gene transfer [21]. Its conserved region has been leveraged for developing PCR assays while its variable region has been used to differentiate *Babesia* species and resolve phylogenetic relationships among species [22]. 

Meanwhile, the apical complex, which includes secretory organelles rhoptries, micronemes, and dense granules (analogous to spherical bodies in *Babesia* and *Theileria*), is a defining structural characteristic in all apicomplexan parasites [23]. In *Babesia*, these three organelles secrete proteins that are involved in parasite attachment, invasion, and post-invasion host cell modifications [24]. *Babesia* rhoptry-associated protein 1 (RAP-1) is a variable multigene family which is characterized by very minimal intraspecies diversity and relatively high interspecies diversity [25]. *Babesia bovis rap-1* sequences and *B. bigemina rap-1a* sequences were shown to be highly conserved, demonstrating the strongpoint of *rap-1* gene as a diagnostic marker in epidemiological surveys [26,27]. In a similar manner, the apical membrane antigen (AMA-1) is an essential protein implicated in the erythrocyte invasion of the parasite [24]. In previous investigations, the *ama-1* gene proved to be greatly conserved among various geographical isolates, making it an invaluable diagnostic target for parasite detection [26,28,29,30,31]. On the other hand, spherical body proteins (SBP) are involved in alterations and remodeling of the infected erythrocytes and are localized in the spherical body organelles post-invasion [24]. PCR assays developed based on the *sbp-2* and *sbp-4* genes have been widely used to detect *B. bovis* in field samples from different parts of the world [32,33].

## 4. Molecular Reports of *Babesia* in Ruminants in Southeast Asia

### 4.1. Bovine Babesiosis

The world cattle population stands at 1.5 billion heads, of which a little below one-third of the population is raised in the Asian continent [7]. Compared to other subregions, cattle in SEA account for a relatively minute portion of the total cattle population in Asia. Despite this, SEA’s total bovine production is still considered a significant contributor to meeting the exponentially rising demand for cattle produce. In 2019, Southeast Asian bovine production consisted of 1.74 million tons of beef, 5.57 million tons of cow milk, 237,000 tons of hide, and 55,000 tons of fat [7].

**Table 3:** Molecular reports of *Babesia* in cattle in Southeast Asian countries.

Country	Pathogen	Conventional PCR			Nested PCR		
		Detection Rate (%) *	Samples (*n*)	References	Detection Rate (%) *	Samples (*n*)	References
Vietnam	*Babesia bigemina*	5.20–22.60	96–258	[20,41,42,43]	16.00	94	[44]
	*Babesia bovis*	4.20–12.30	120–258	[20,43,45]	15.60–21.30	94–96	[41,44]
	*Babesia* sp. Hue	1.20	258	[42]	n. r.		
	*Babesia ovata*	0.00	184	[29]	n. r.		
	*Babesia* sp. Mymensingh	9.60	460	[30]	n. r.		
Philippines	*Babesia bigemina*	15.40–61.70	339–408	[46,47]	0–10.80	48–412	[48,49,50,51]
	*Babesia bovis*	10.00–45.40	339–408	[46,47]	0–11.50	48–412	[48,49,50,51]
	*Babesia ovata*	0.00	300	[29]	n. r.		
	*Babesia* sp. Mymensingh	11.30	408	[30]	n. r.		
	*Babesia* spp.	2.00	246	[52]	n. r.		
Thailand	*Babesia bigemina*	n. r.			2.90–38.90	96–329	[34,53,54,55,56]
	*Babesia bovis*	n. r.			1.40–24.50	53–1824	[34,53,54,55,57]
	*Babesia ovata*	2.50	200	[29]	n. r.		
Indonesia	*Babesia bigemina*	14.20	141	[58]	19.10	487	[59]
	*Babesia bovis*	34.80	141	[58]	50.70	487	[59]
Myanmar	*Babesia bigemina*	9.80	713	[60]	n. r.		
	*Babesia bovis*	n. r.			17.10	713	[60]
Malaysia	*Babesia bigemina*	30.50	1,045	[61,62]	n. r.		
	*Babesia bovis*	32.50	1045	[62]	n. r.		

* Total detection rates from each study were used. n. r.: no report.

Among the domestic ruminants covered in this mini-review, babesiosis in cattle is more extensively studied in SEA, owing to the well-known susceptibility of cattle to the disease, specifically those of the taurine breed [63]. Twenty years ago, an economic assessment of the impact of cattle fever (babesiosis and anaplasmosis) estimated herd mortality rates of 0.5% in Indonesia, 0.1% in the Philippines, and 0.5% in Thailand [64]. Furthermore, bovine production losses amounting to USD 3.10 million and USD 0.60 million were calculated for Indonesia and the Philippines, respectively [64].

The *Babesia* species that are known to infect cattle are *Babesia bovis*, *B*. *bigemina*, *B. major*, *B*. *divergens*, *B*. *ovata*, *B*. *occultans*, *B*. *jakimovi* [65], and several undescribed taxa, namely *Babesia* sp. Oshima [66], *Babesia* sp. Kashi [67], *Babesia* sp. Hue [42], and *Babesia* sp. Mymensingh [68]. *Babesia bovis* and *B*. *bigemina* are the most commonly reported etiologic agents of bovine babesiosis worldwide and have the greatest impact on bovines [69]. These two species are widely present in tropical and subtropical regions where the tick vectors *Rhipicephalus* and *Ixodes* are present. Cattle infected with *B. bovis* can be severely ill compared to the milder *B. bigemina* infection [3]. On the other hand, the predominant bovine *Babesia* in Europe includes the zoonotic *B. divergens* [70] and the less pathogenic *B. major* [1]. Additionally, *B. occultans* and *B. ovata* were thought to have low pathogenicity in cattle [1,71], but clinical outbreaks [72,73] and cases of exacerbated anemia [38] have been attributed to each respective species. Of the undescribed species, only *Babesia* sp. Mymensingh has been proven to be of major clinical significance [39]. 

Hitherto, five *Babesia* species, specifically *B. bigemina*, *B. bovis*, *B. ovata*, *Babesia* sp. Hue, and *Babesia* sp. Mymensingh, have been identified in SEA after molecular screening of more than seven thousand individual samples as reported in 25 molecular studies conducted in cattle (Figure 1 and Table S1). Countries with the most numbers of cattle surveyed were Thailand (*n* = 2929), the Philippines (*n* = 1851), and Malaysia (*n* = 1045). The species *B. bovis* and *B. bigemina* were detected in bovine blood DNA samples collected from Indonesia, Malaysia, Myanmar, the Philippines, Thailand, and Vietnam (Table 3), with detection rates as high as 61.70% (cPCR) for *B. bigemina* [46] and 50.70% (nPCR) for *B. bovis* [59]. Meanwhile, *B. ovata* investigations were conducted in three countries, but its presence was detected only in Thailand [29] and Vietnam [42] (Table 3). Interestingly, sequences of *B. ovata*-positive cattle samples in Vietnam led to the discovery of a *B. ovata*-related benign species designated as *Babesia* sp. Hue [42]. In addition, upon the comprehensive description of the novel species *Babesia* sp. Mymensingh in cattle, a molecular survey detected the parasite in 11.30% (cPCR) and 9.57% (cPCR) of archived cattle DNA samples from the Philippines and Vietnam, respectively [30].

### 4.2. Bubaline Babesiosis

The majority of the world’s 198 million water buffaloes are found in Asia [7]. About 70% of the bubaline population of SEA is concentrated in Myanmar, the Philippines, and Vietnam [7], most of which are largely owned by small-scale farmers for draft work in unmechanized crop production systems and as means of transportation in the rural areas [74]. Besides these, buffalo raising can be a source of additional income in the form of milk and meat and breeding stock. Moreover, the water buffalo’s sturdiness and rusticity enable farmers to keep them with minimal sustenance costs based on low-quality fodder. 

Similar to cattle, *B. bovis* and *B. bigemina* are the primary species affecting buffaloes. In contrast to the more obvious signs in cattle, clinical babesiosis in water buffaloes is rare and has been clinically documented only with *B. bigemina* infections [75]. This relatively stronger resistance of water buffaloes to developing clinical disease after *B. bovis* infection was also observed experimentally [76]. The prevailing hypothesis posed by Benitez et al. [76] is largely based on the probable co-evolutionary adaptation among *B. bovis* –buffalo–*Rhipicephalus* ticks, which could explain the resistance of water buffaloes to pathogenic *B. bovis*. Another species, *B. orientalis*, is known to be pathogenic in water buffalo and occurs only in the southeastern part of China [77].

Compared to cattle surveys, molecular studies in water buffaloes in SEA are notably fewer (Figure 1 and Table 4). A total of 1156 (*n*) individual water buffalo samples from Indonesia, the Philippines, Thailand, and Vietnam have been molecularly evaluated for various *Babesia* species (Table 4 and Table S2). The highest detection rate for bubaline *B. bovis* was 32.70% (cPCR) in Vietnam, 21.10% (cPCR) in Indonesia, 21.00% (nPCR) in the Philippines, and 11.20% (nPCR) in Thailand. In the case of *B. bigemina*, the highest detection rates were 17.50% (cPCR), 4.40% (cPCR), 4.10% (cPCR), and 3.60% (nPCR) in Indonesia, the Philippines, Vietnam, and Thailand, respectively. Finally, the detection of *Babesia* sp. Mymensingh in samples from Vietnam (Table 4) added water buffalo to the list of host ranges of this novel *Babesia* species [30].

**Table 4:** Molecular reports of *Babesia* in water buffaloes in Southeast Asian countries.

Country	Pathogen	Conventional PCR			Nested PCR		
		Detection Rate (%) *	Samples (*n*)	References	Detection Rate (%) *	Samples (*n*)	References
Vietnam	*Babesia bigemina*	0–4.10	43–49	[41,42]	0	43	[44]
	*Babesia bovis*	32.70	49	[45]	9.30–23.30	43; 43	[41,44]
	*Babesia ovata*	0	49	[42]	n. r.		
	*Babesia* sp. Mymensingh	2.30–18.40	43–49	[30]	n. r.		
Philippines	*Babesia bigemina*	4.40	272	[78]	0–3.00	65–114	[49,50,51,79]
	*Babesia bovis*	n. r.			0–21.00	65–114	[49,50,51,79]
	*Babesia ovata*	0	100	[79]	n. r.		
Thailand	*Babesia bigemina*	n. r.			3.60	305	[33]
	*Babesia bovis*	n. r.			11.20	305	[33]
Indonesia	*Babesia bigemina*	17.50	57	[58]	n. r.		
	*Babesia bovis*	21.10	57	[58]	n. r.		

* Total detection rates from each study were used. n. r.: no report.

### 4.3. Caprine and Ovine Babesiosis

Sheep and goats are among the earliest domesticated animals by humans, preceding cattle domestication by thousands of years [80]. There are over 1.2 billion sheep and 1 billion goats in the world [7]. Sheep and goat production has a significant socioeconomic value for rural households and subsistence farming families, specifically as a supplement to farmers’ income and as means of additional food sources [81]. However, herd health is often neglected despite the high susceptibility of small ruminants to major infections such as those caused by parasitic helminths, arthropods, and protozoa [82,83], which include TBDs such as babesiosis and theileriosis. 

Babesiosis causes huge economic losses in terms of lower production of milk, meat, and other livestock byproducts, combined with the indirect burden of additional cost for treatment of animals, control of the disease, and the opportunity cost of production [3]. Various species of *Babesia* are responsible for causing babesiosis in small ruminants, including *B. ovis*, *B. motasi*, *B. crassa*, *B. motasi*-like, and *Babesia* sp. Xinjiang [69,84]. Of these causative agents of babesiosis, *B. ovis* is the most severely pathogenic species and is responsible for causing fever, hemoglobinuria, anemia, and icterus, oftentimes leading to death [85]. In the field, mortality caused by *B. ovis* infection ranges from 30% to 50% in sheep [86], while natural infection in goats is subclinical [87]. *Babesia motasi* may have milder virulence in sheep but is more common in goats [88], whereas *B. crassa* seems to have low pathogenicity [89]. Ixodid ticks belonging to the genus *Rhipicephalus* and *Hyalomma* are the vectors of *B. ovis*, while *B. motasi* is transmitted by *Haemaphysalis* and *Rhipicephalus* ticks [90]. *Babesia ovis* is widely distributed globally, while other ovine and caprine *Babesia* species occur only in particular areas [88]. Although believed to be present, the distribution of *B. ovis* in SEA has been sporadic and its occurrence is generally unknown [90]. 

Despite goats and sheep ranking second (37 million) and third (19 million) in terms of the population of all ruminants in SEA, only a small number of goats and sheep have been molecularly evaluated for babesiosis (Table 5 and Table S3). So far, a total of six molecular investigations on small ruminant babesiosis in SEA have been conducted (Table 5). *Babesia ovis* was recently confirmed in Philippine goats [91], while *Babesia* sp. was molecularly detected in goats in Thailand [92]. In Vietnam, goats and sheep were positive for *Babesia* sp. Mymensingh, further expanding the host range of this species, whereas *B. bigemina* DNA was detected in a goat sample [41]. 

**Table 5:** Molecular detection rates for *Babesia* in small ruminants in Southeast Asian countries.

Country	Host	Pathogen	Detection Rate (%) *	Samples (*n*)	References
Vietnam	goat	*Babesia bigemina*	0.80	127	[41]
	sheep	*Babesia bigemina*	0	51	[41]
	goat	*Babesia bovis*	0	127	[41]
	sheep	*Babesia bovis*	0	51	[41]
	goat	*Babesia* sp. Mymensingh	1.60	127	[30]
	sheep	*Babesia* sp. Mymensingh	2.00	51	[30]
Philippines	goat	*Babesia ovis*	1.50	396	[91]
	goat	*Babesia* spp.	0	100	[93]
Thailand	goat	*Babesia* spp.	2.00	100	[92]
	goat	*Babesia ovis*	0	262	[94]

* Total detection rates from each study were used.

## 5. Factors Associated with Ruminant *Babesia* Infection in SEA

Several factors have been associated with bovine babesiosis in SEA. Studies conducted in Thailand [53], Myanmar [60], and Malaysia [61] identified a higher number of young cattle that tested positive for bovine *Babesia*, whereas cattle age was negligible in bovine *Babesia* infections reported in the Philippines [48] and Indonesia [59]. In cattle, inverse age immunity, where the development of clinical disease is low, is an observed characteristic of bovine babesiosis and anaplasmosis in endemically stable areas. Young animals are exposed to the infection early in their life when they have a more robust immunity through maternal antibodies and strong innate immunity, enabling them to acquire natural protection against subsequent infections [13]. Likewise, higher *B. bovis* infection rates were recorded for taurine breeds and/or crossbreds compared with common indicine breeds (i.e., Zebu, Brahman) in Myanmar [60] and Indonesia [59]. A similar trend for *B. bigemina* infection was observed in cattle in Malaysia [61]. The impact of babesiosis on *Bos indicus* cattle is known to be milder compared with that on *Bos taurus* [63]. Notably, the indigenous breeds in Indonesia recorded higher molecular detection rates of *B. bovis* (Bali cattle) and of *B. bigemina* (Pesisir cattle) [59], suggesting that other cattle breeds may have variable susceptibility to *Babesia* infections. On the other hand, the sex of cattle was not associated with bovine *Babesia* positivity in surveys in the Philippines [48], Thailand [53], Myanmar [60], and Malaysia [61]. Additionally, the practice of grazing has been identified as a significant factor for bovine *Babesia* infections in Thailand [54], *B. bovis* infection in Myanmar [60], and *B. bigemina* infection in Malaysia [61]. The extensive management system may be directly linked to the increased exposure of the animals to the vectors that may carry the parasites.

Studies that evaluated significant factors for *Babesia* infection in water buffaloes in SEA are scarce. In Thailand, the age of the animal was associated with *B. bovis* or *B. bigemina* positivity [33], while the opposite was observed in the Philippines [79]. Furthermore, *Babesia* infections did not differ between sexes and among breeds in water buffaloes in Thailand and the Philippines, respectively [33,79]. Meanwhile, as *Babesia* detection studies in SEA small ruminants are in their infancy, risk factors related to such are virtually non-existent. Therefore, identifying significant factors that may increase the risk of water buffaloes, goats, and sheep to contract babesiosis may be a valuable topic to explore in future investigations.

## 6. Conclusions

In this mini-review, we compiled the existing molecular records and mapped the species diversity of *Babesia* in large and small ruminants in SEA. Molecularly confirmed *Babesia* species in Southeast Asian ruminants include *B. bovis*, *B. bigemina*, *B. ovata*, *B. ovis*, *Babesia* sp. Hue, and *Babesia* sp. Mymensingh. To date, molecular studies in cattle and water buffaloes have provided fundamental information on babesiosis, whereas studies on small ruminants are lacking and need more attention considering that small ruminant production is a common venture among many rural farming communities in SEA. 

In an epidemiological context, molecular babesiosis research in some SEA countries has had significant success in confirming the presence of various *Babesia* species, albeit, it has been inadequate in truly uncovering the situation of ruminant babesiosis in the field. This calls for more extensive molecular surveillance, particularly in countries with denser ruminant populations. With various molecular diagnostic platforms becoming relatively more affordable and accessible, their utility in *Babesia* infection diagnosis in the field has been beneficial and shall play an important part in assessing the disease’s real impact on animal production and in formulating and implementing control programs for economically devastating diseases such as babesiosis and other TBDs.

## Figures and Tables

**Table 1:** Ruminant population in Southeast Asian countries as of 2019.

Country	Cattle	Water Buffalo	Goat	Sheep	Ruminant Population per Country
Brunei Darussalam	617	2292	1016	4649 *	8574
Cambodia	2,848,846 *	605,638 *	n. a.	n. a.	3,454,484
Indonesia	17,118,650	1,141,298	18,975,955	17,794,344	55,030,247
Laos	2,092,344 *	1,209,712 *	639,715 *	n. a.	3,941,771
Malaysia	683,501	107,347	371,747	127,796	1,290,391
Myanmar	18,583,932 *	4,082,914 *	10,940,257 *	1,309,307 *	34,916,410
Philippines	2,535,414	2,873,561	3,755,879	30,000	9,194,854
Singapore	169 *	n. a.	755 *	n. a.	924
Thailand	4,600,000 ^#^	897,368 *	478,559 *	39,662 *	6,015,589
Timor-Leste	213,235 *	126,066 *	66,504 *	42,593 *	448,398
Vietnam	6,060,024	2,387,887	2,609,198	n. a.	11,057,109
Total Ruminant Population in Southeast Asia	54,736,732	13,434,083	37,839,585	19,348,351	125,358,751

Population inventories were derived from the Food and Agriculture Organization Corporate Statistical Database (FAOSTAT) [7]. * Data were calculated based on imputation method; ^#^ estimated data. Abbreviation—n. a.: not available.

## Data Availability

New data were not generated in the current study; thus, statement of data availability is not applicable.

## Supplementary Materials

The following are available online at https://www.mdpi.com/article/10.3390/pathogens11080915/s1, Table S1. List of PCR-based *Babesia* reports on cattle in Southeast Asia. Table S2. List of PCR-based *Babesia* reports on water buffaloes in Southeast Asia. Table S3. List of PCR-based *Babesia* reports on small ruminants in Southeast Asia.
